# Supplementary material for: Virtual Care in Patients with Cancer: A Systematic Review
Source: Curr Oncol. 2021 Sep 8;28(5):3488–506. doi: 10.3390/curroncol28050301 (PMC8482228; doi:10.3390/curroncol28050301)
Supplement: Supplementary file 1 [file curroncol-28-00301-s001.zip › curroncol-1352334-supplementary.pdf]

Systematic Review

Virtual Care in Patients with Cancer: A Systematic Review

Simron Singh [1], Glenn G. Fletcher [2], Xiaomei Yao [2,3], Jonathan Sussman [2,*]

Supplementary Material

Table S1. Literature Search Strategy (Databases).

(1) Database(s): Embase 1996 to 2020 August 06, APA PsycInfo 1987 to July Week 4 2020, EBM Reviews - Cochrane Central Register of Controlled Trials June 2020, EBM Reviews - Cochrane Database of Systematic Reviews 2005 to July 31, 2020, Ovid MEDLINE(R) and Epub Ahead of Print, In-Process & Other Non-Indexed Citations, Daily and Versions(R) 1946 to August 06, 2020

#	Searches	Results
1	exp Telemedicine/ or Remote Consultation/ or exp Teleconsultation/ or exp Telerehabilitation/ or exp Telehealth/	86831
2	exp Consultation/ or exp Disease Management/ or exp Patient Monitoring/ or exp Follow up/ or exp Patient Care/ or exp "Evaluation and Follow up"/ or exp Medical Examination/ or exp Physical Examination/	7619641
3	exp Mobile Phone/ or exp Cell Phone/ or exp Mobile Application/ or exp Smartphone/ or exp Teleconference/ or exp Videoconferencing/	153534
4	1 or (2 and 3)	120953
5	(Telemedicine or Remote Consultation or Teleconsultation or Telerehabilitation or Telehealth or eHealth or e-Health or mHealth or m-Health or E-oncology).ti,ab.	60684
6	((virtual or distance or distant or remote or video or phone or smartphone or cellphone or cell phone or mobile or digital or teleconferenc: or videoconferenc: or computer or internet or online) adj3 (consult: or manage: or monitor: or follow-up or care or evaluat: or exam: or treat: intervene or intervention: or assess: or diagnos: or navigat: or "after-care" or deliver: or service: or contact:)).ti,ab.	275535
7	4 or 5 or 6	389680
8	exp Neoplasms/ or exp Neoplasm/ or exp Tumor/ or exp Cancer/ or (Cancer: or Tumor: or Tumour: or Neoplas: or Oncolog: or Metasta: or Malignan: or Carcinom: or Andenocarcinom: or Leukemia: or Leukaemia: or Lymphoma: or Sarcoma: or Melanoma:).ti,ab.	9350508
9	7 and 8	51349
10	limit 9 to yr="2015 -Current"	23160
11	10 not (abstract or "conference abstract" or news: or case report:).pt.	17165
12	limit 11 to english language [Limit not valid in CDSR; records were retained]	15698
13	limit 11 to no language specified [Limit not valid in APA PsycInfo,CDSR; records were retained]	1498
14	limit 11 to French [Limit not valid in CDSR; records were retained]	89
15	12 or 13 or 14	16665
16	limit 15 to yr="2015-2016"	4705
17	limit 15 to yr="2017-2018"	5876
18	limit 15 to yr="2019"	3166
19	16 or 17 or 18	13747
20	15 not 19	2918
21	remove duplicates from 16	2921
22	remove duplicates from 17	3703

23	remove duplicates from 18	2025
24	remove duplicates from 20	1803
25	21 or 22 or 23 or 24	10452

(2) Database(s): EBM Reviews - Cochrane Database of Systematic Reviews 2005 to August 1, 2020 Search Strategy:

#	Searches	Results
1	(Telemedicine or Remote Consultation or Teleconsultation or Telerehabilitation or Telehealth or eHealth or e-Health or mHealth or m-Health or E-oncology).ti,ab.	23
2	((virtual or distance or distant or remote or video or phone or smartphone or cellphone or cell phone or mobile or digital or teleconferenc: or videoconferenc: or computer or internet or online) adj3 (consult: or manage: or monitor: or follow-up or care or evaluat: or exam: or treat: intervene or intervention: or assess: or diagnos: or navigat: or "after-care" or deliver: or service: or contact:)).ti,ab.	116
3	(Cancer: or Tumor: or Tumour: or Neoplas: or Oncolog: or Metasta: or Malignan: or Carcinom: or Andenocarcinom: or Leukemia: or Leukaemia: or Lymphoma: or Sarcoma: or Melanoma:).ti,ab.	1279
4	1 or 2	130
5	3 and 4	16

(3) CINAHL: July 29, 2020

	Search Terms	Actions
S1	(MH "Telehealth") OR (MH "Telemedicine+") OR (MH "Telerehabilitation") or (MH "Telenursing") or (MH "Remote Consultation")	(6,890)
S2	(((virtual or distance or distant or remote or video or phone or smartphone or cellphone or cell phone or mobile or digital or teleconferenc: or videoconferenc: or computer or internet or online) N3 (consult: or manage: or monitor: or follow-up or care or evaluat: or exam: or treat: intervene or intervention: or assess: or diagnos: or navigat: or "after-care" or deliver: or service: or contact:)) OR AB (((virtual or distance or distant or remote or video or phone or smartphone or cellphone or cell phone or mobile or digital or teleconferenc: or videoconferenc: or computer or internet or online) N3 (consult: or manage: or monitor: or follow-up or care or evaluat: or exam: or treat: intervene or intervention: or assess: or diagnos: or navigat: or "after-care" or deliver: or service: or contact:))	(6,013)
S3	TI ((Telemedicine or Remote Consultation or Teleconsultation or Telerehabilitation or Telehealth or eHealth or e-Health or mHealth or m-Health or E-oncology)) OR AB ((Telemedicine or Remote Consultation or Teleconsultation or Telerehabilitation or Telehealth or eHealth or e-Health or mHealth or m-Health or E-oncology))	(4,716)
S4	((MH "Oncologic Nursing+") OR (MH "Oncologic Care+") OR (MH "Cancer Patients") OR (MH "Oncology+") OR (MH "Neoplasms+")) OR TI ((Cancer: or Tumor: or Tumour: or Neoplas: or Oncolog: or Metasta: or Malignan: or Carcinom: or Andenocarcinom: or Leukemia: or Leukaemia: or Lymphoma: or Sarcoma: or Melanoma:)) OR AB ((Cancer: or Tumor: or Tumour: or Neoplas: or Oncolog: or Metasta: or Malignan: or Carcinom: or Andenocarcinom: or Leukemia: or Leukaemia: or Lymphoma: or Sarcoma: or Melanoma:))	(135,700)
S5	S1 OR S2 OR S3	(13,959)
S6	S4 AND S5	(790)

Above is limited to 2015- 2020, and excludes citations from Medline.

(4). Search terms for ongoing trials on clinicaltrials.gov included in-person, virtual care, telemedicine, and telephone (one term each time).

Table S2. Ongoing trials.

Author and source; trial name; recruitment or follow-up period; estimated study completion date	# Patients anticipated	Patient characteristics	Trial type and comparison	Outcome
Chau, 2019 [1]<br>REACH PC<br>NCT03375489<br>Massachusetts General Hospital + 19 other sites<br>2018-2022 (estimated)<br>Estimated completion May 31 2023	1250 patients<br>Up to 1250 caregivers if patient chose to invite them	Advanced non-small cell lung cancer within 12 weeks of diagnosis and being treated with non-curative intent.<br>Recruited through 20 Palliative Care Research Cooperative institutions in USA.<br>Patients may invite caregiver (family or friend who lives with them or contact at least twice a week) to participate with them.	RCT.<br>Telehealth by videoconferencing early palliative care (monthly) vs. in-person early palliative care (monthly) by specialty-trained experienced or board-certified PC physicians or advanced practice nurses who provide care in the outpatient oncology setting. Initial visit for both groups is in-person	QoL, mood, and quality of communication with oncologists at baseline before randomization and at 12, 24, 36, and 48 weeks<br>Health care utilization, including length of stay in hospice, will be collected from patients' health records
Telemedicine Nurse-Led Intervention for Rural Cancer Survivors (CARING) [2]<br>NCT04267627<br>University of Virginia<br>2020-2025 (estimated)<br>Estimated completion January 1, 2026	450	Patients with head and neck cancer at any stage who completed treatment within the last 6 weeks or anticipated to be within 3 months of end of treatment<br>Plan to oversample rural patients	RCT (3 arm)<br>Nurse-lead supportive care (CARING) by telemedicine videoconferencing vs. CARING in-person vs. usual care	Unmet needs, QoL (FACT-HN)<br>Cost effectiveness<br>Patient perceptions of telemedicine
Telehealth for palliative care patients in metropolitan and rural settings [3]<br>ACTRN12618001007224<br>HREC/18/MonH/348<br>2018-2020 (anticipated)<br>Monash, Victoria, Australia<br>Estimated completion date not reported	140	Patients admitted to an associated community palliative care service, a Monash Health inpatient palliative care unit, or engaged with a Monash Health palliative care consult service or the Monash Health Oncology Supportive Care Outpatient Clinic.<br>Diagnosed with life-limiting illness (cancer, cardiovascular, musculoskeletal, neurological)	RCT.<br>Telehealth videoconference with palliative care physician and nurse facilitation at 2 weeks then as required for 3 months vs. standard care (usual care provided including community palliative care, other community services, hospital services, specialists, and general practice)	Clinical symptoms (sleep, nausea, pain, fatigue, breathing problems, bowel problems), QoL, Performance Status (Karnofsky),<br>Secondary outcomes: emergency department attendances, teleconference efficiency (setup), user experience, health professional score of symptom severity (pain, other symptoms, psycho-spiritual distress, carer distress), QoL (family), Hospital Anxiety and Depression Scale, hospital admissions, home visit duration

Author and source; trial name; recruitment or follow-up period; estimated study completion date	# Patients anticipated	Patient characteristics	Trial type and comparison	Outcome
Supportive Care Delivered by Telemedicine to Cancer Patients at Home [4] NCT04136340 2019-2021 (estimated) Rockefeller Outpatient Pavilion, Memorial Sloan Kettering Cancer Center, New York Estimated completion October, 2023	466	Cancer; planned for Supportive Care Service, resident of New York, New Jersey, or Connecticut	RCT, non-inferiority pilot trial Supportive palliative care specialist in –person for first visit then follow-up care by in-home video telemedicine vs. in-person at clinic. At least 3 follow-up visits over 14 weeks	Health-related QoL (FACT-G) at baseline and week 14
Assessing the System for High-Intensity Evaluation During Radiotherapy During Changes in Response to COVID-19 (CORONA-SHIELD) [5] NCT04357574 Duke Cancer Center, Durham, North Carolina, USA 2020-2021 (estimated) Estimated completion April 2022	1000	Patients undergoing radiation oncology during COVID-19 outbreak; no concurrent systemic therapy	Observational study Patients during COVID-19 outbreak and their healthcare provider experience Comparison of rates of acute care for telehealth (telephone or video) vs. prior rates	Healthcare provider perceptions of telehealth Rates of acute care (emergency department visits and hospitalizations)

Table S3. Summary Table for Risk of Bias Assessment for Randomized Controlled Trials.

Study	Outcome	Domain 1: Randomization Process	Domain 2: Deviation from Intervention	Domain 3: Missing Outcome Data	Domain 4: Measurement of Outcome	Domain 5: Reported Results	Overall Risk of Bias	
							Per outcome	Per study
Lleras de Frutos, 2020 [6]	Emotional distress	Some concerns	Some concerns	High	Low	Low	High	High
	Post-traumatic stress	Some concerns	Some concerns	High	Low	Low	High	
	Post-traumatic growth	Some concerns	Some concerns	High	Low	Low	High	
Watson, 2017 [7]	Anxiety and depression	Low	Some concerns	High	Low	Low	High	High
	Mental adjustment to cancer	Low	Some concerns	High	Low	Low	High	
	Cancer concerns	Low	Some concerns	High	Low	Low	High	
	Cancer coping questionnaire	Low	Some concerns	High	Low	Low	High	
Harrigan, 2016 [8]	Body composition	Some concerns	High	High	Low	Low	High	High
	Physical activity	Some concerns	High	High	Low	Low	High	
	Diet	Some concerns	High	High	Low	Low	High	
	Serum biomarkers	Some concerns	High	High	Low	Low	High	
Buchanan, 2015 [9]; Datta, 2011 [10]	Cost	Low	Low	Low	Low	Low	Low	High
	Satisfaction	Some concerns	High	Low	Low	Low	High	
	Attendance	Some concerns	High	Low	Low	Low	High	
Steffen, 2017 [11]; Kinney, 2016 [12]; Chang, 2016 [13]	Psychosocial	Low	Some concerns	Low	Low	Low	Some concerns	High
	Genetic testing uptake	Low	Some concerns	High	Low	Low	High	
	Counselling moderators of genetic testing[1]	Low	Some concerns	High	Low	Low	High	
	Cost	Low	Low	Low	Low	Low	Low	
Jacobs, 2016 [14]; Peshkin, 2016 [15]; Schwartz, 2014 [16];	Knowledge	Low	Low	Low	Low	Low	Low	Low
	Patient satisfaction	Low	Low	Low	Low	Low	Low	
	Decision conflict	Low	Low	Low	Low	Low	Low	

[1] Cancer-specific distress, perceived risk of a genetic mutation compared to population risk, counselor patient-centeredness, cost barriers, decisional conflict, education, social support, family history of cancer

Butrick, 2015 [17]	Distress	Low	Low	Low	Low	Low	Low	
	Genetic counsellor questionnaire	Low	Low	Low	Low	Low	Low	
Kelleher, 2019 [18]; Winger, 2020 [19]	Access[2]	Low	High	Some concerns	Low	Low	High	High
	Pain[3]	High	High	Some concerns	Low	Some concerns	High	
	Symptoms, distress[4]	High	High	Some concerns	Low	Some concerns	High	
Walle, 2018, 2020 [20,21]	Feasibility	Low	Low	Low	Low	Low	Low	High
	Patient cost	Some concerns	Some concerns	Some concerns	Low	Low	High	
	Patient time	Some concerns	Some concerns	Some concerns	Low	Low	High	
	Satisfaction	Some concerns	Some concerns	Some concerns	Low	Low	High	
	Physician-patient relationship	Some concerns	Some concerns	Some concerns	Low	Low	High	
Beaver, 2017 [22]; Dixon, 2018 [23]; Beaver, 2020 [24]	Psychological morbidity	Low	Low	Low	Low	Low	Low	Low
	Satisfaction	Low	Low	Low	Low	Low	Low	
	Quality of life	Low	Low	Low	Low	Low	Low	
	Recurrence	Low	Low	Low	Low	Low	Low	
Viers, 2015 [25]	Efficiency (duration of visit + waiting)	Some concerns	Some concerns	Some concerns	Some concerns	Low	High	High
	Satisfaction	Some concerns	Some concerns	Some concerns	Some concerns	Low	High	
	Patient cost	Some concerns	Some concerns	Some concerns	Some concerns	Some concerns	High	

[2] Aim 1: intervention access (feasibility [attrition, adherence, completion time], patient burden [physical, emotional, financial], engagement, acceptability)

[3] Aim 2: primary outcomes: pain severity and pain interference

[4] Aim 2: secondary outcomes: physical well-being and symptoms, psychological distress, self-efficacy for pain management

References:

1. Chua, I.S.; Zachariah, F.; Dale, W.; Feliciano, J.; Hanson, L.; Blackhall, L.; Quest, T.; Curseen, K.; Grey, C.; Rhodes, R.; et al. Early integrated telehealth versus in-person palliative care for patients with advanced lung cancer: A study protocol. *J Palliat Med* **2019**, *22*, 7-19, doi:http://dx.doi.org/10.1089/jpm.2019.0210.
2. National Library of Medicine (US). Telemedicine nurse-led intervention for rural cancer survivors. *https://clinicaltrials.gov/show/NCT04267627* **2020**.
3. Australian and New Zealand Clinical Trials Registry [Internet]. Telehealth for palliative care patients in metropolitan and rural settings. Sydney (NSW): NHMRC Clinical Trials Centre, University of Sydney (Australia), 2018. Available from: https://anzctr.org.au/Trial/Registration/TrialReview.aspx?ACTRN=12618001007224 (assessed Aug 7, 2020). **2018**.
4. National Library of Medicine (US). Supportive care delivered by telemedicine to cancer patients at home. *https://clinicaltrials.gov/show/NCT04136340* **2019**.
5. National Library of Medicine (US). Assessing the system for high-intensity evaluation during radiotherapy during changes in response to COVID-19 (CORONA-SHIELD). https://clinicaltrials.gov/ct2/show/NCT04357574. **2020**.
6. Lleras de Frutos, M.; Medina, J.C.; Vives, J.; Casellas-Grau, A.; Marzo, J.L.; Borras, J.M.; Ochoa-Arnedo, C. Video conference vs face-to-face group psychotherapy for distressed cancer survivors: A randomized controlled trial. *Psychooncology* **2020**, *03*, 03, doi:http://dx.doi.org/10.1002/pon.5457.
7. Watson, M.; White, C.; Lynch, A.; Mohammed, K. Telephone-delivered individual cognitive behavioural therapy for cancer patients: An equivalence randomised trial. *Psychooncology* **2017**, *26*, 301-308, doi:http://dx.doi.org/10.1002/pon.4338.
8. Harrigan, M.; Cartmel, B.; Loftfield, E.; Sanft, T.; Chagpar, A.B.; Zhou, Y.; Playdon, M.; Li, F.; Irwin, M.L. Randomized trial comparing telephone versus in-person weight loss counseling on body composition and circulating biomarkers in women treated for breast cancer: The Lifestyle, Exercise, and Nutrition (LEAN) Study. *J Clin Oncol* **2016**, *34*, 669-676, doi:http://dx.doi.org/10.1200/JCO.2015.61.6375.
9. Buchanan, A.H.; Datta, S.K.; Skinner, C.S.; Hollowell, G.P.; Beresford, H.F.; Freeland, T.; Rogers, B.; Boling, J.; Marcom, P.K.; Adams, M.B. Randomized trial of telegenetics vs. in-person cancer genetic counseling: Cost, patient satisfaction and attendance. *J Genet Couns* **2015**, *24*, 961-970, doi:https://dx.doi.org/10.1007/s10897-015-9836-6.
10. Datta, S.K.; Buchanan, A.H.; Hollowell, G.P.; Beresford, H.F.; Marcom, P.K.; Adams, M.B. Telemedicine vs in-person cancer genetic counseling: Measuring satisfaction and conducting economic analysis. *Comp Eff Res (Auckl)* **2011**, *1*, 43-50, doi:http://dx.doi.org/10.2147/CER.S19179.
11. Steffen, L.E.; Du, R.; Gammon, A.; Mandelblatt, J.S.; Kohlmann, W.K.; Lee, J.H.; Buys, S.S.; Stroup, A.M.; Campo, R.A.; Flores, K.G.; et al. Genetic testing in a population-based sample of breast and ovarian cancer survivors from the REACH randomized trial: Cost barriers and moderators of counseling mode. *Cancer Epidemiol Biomarkers Prev* **2017**, *26*, 1772-1780, doi:http://dx.doi.org/10.1158/1055-9965.EPI-17-0389.
12. Kinney, A.Y.; Steffen, L.E.; Brumbach, B.H.; Kohlmann, W.; Du, R.; Lee, J.H.; Gammon, A.; Butler, K.; Buys, S.S.; Stroup, A.M.; et al. Randomized noninferiority trial of telephone delivery of BRCA1/2 genetic counseling compared with in-person counseling: 1-year follow-up. *J Clin Oncol* **2016**, *34*, 2914-2924, doi:http://dx.doi.org/10.1200/JCO.2015.65.9557.
13. Chang, Y.; Near, A.M.; Butler, K.M.; Hoeffken, A.; Edwards, S.L.; Stroup, A.M.; Kohlmann, W.; Gammon, A.; Buys, S.S.; Schwartz, M.D.; et al. Economic evaluation alongside a clinical trial of telephone versus in-person genetic counseling for BRCA1/2 mutations in geographically underserved areas. *J Oncol Pract* **2016**, *12*, e1-e13, doi:http://dx.doi.org/10.1200/JOP.2015.004838.
14. Jacobs, A.S.; Schwartz, M.D.; Valdimarsdottir, H.; Nusbaum, R.H.; Hooker, G.W.; DeMarco, T.A.; Heinzmann, J.E.; McKinnon, W.; McCormick, S.R.; Davis, C.; et al. Patient and genetic counselor perceptions of in-person versus telephone genetic counseling for hereditary breast/ovarian cancer. *Fam Cancer* **2016**, *15*, 529-539, doi:http://dx.doi.org/10.1007/s10689-016-9900-x.
15. Peshkin, B.N.; Kelly, S.; Nusbaum, R.H.; Similuk, M.; DeMarco, T.A.; Hooker, G.W.; Valdimarsdottir, H.B.; Forman, A.D.; Joines, J.R.; Davis, C.; et al. Patient perceptions of telephone vs. in-person BRCA1/BRCA2 genetic counseling. *J Genet Couns* **2016**, *25*, 472-482, doi:10.1007/s10897-015-9897-6.
16. Schwartz, M.D.; Valdimarsdottir, H.B.; Peshkin, B.N.; Mandelblatt, J.; Nusbaum, R.; Huang, A.-T.; Chang, Y.; Graves, K.; Isaacs, C.; Wood, M.; et al. Randomized noninferiority trial of telephone versus in-person genetic counseling for hereditary breast and ovarian cancer. *J Clin Oncol* **2014**, *32*, 618-626, doi:10.1200/JCO.2013.51.3226.
17. Butrick, M.; Kelly, S.; Peshkin, B.N.; Luta, G.; Nusbaum, R.; Hooker, G.W.; Graves, K.; Feeley, L.; Isaacs, C.; Valdimarsdottir, H.B.; et al. Disparities in uptake of BRCA1/2 genetic testing in a randomized trial of telephone counseling. *Genet Med* **2015**, *17*, 467-475, doi:10.1038/gim.2014.125.
18. Kelleher, S.A.; Winger, J.G.; Dorfman, C.S.; Ingle, K.K.; Moskovich, A.A.; Abernethy, A.P.; Keefe, F.J.; Samsa, G.P.; Kimmick, G.G.; Somers, T.J. A behavioral cancer pain intervention: A randomized noninferiority trial comparing in-person with videoconference delivery. *Psychooncology* **2019**, *28*, 1671-1678, doi:http://dx.doi.org/10.1002/pon.5141.
19. Winger, J.G.; Nunez, C.; Kelleher, S.A.; Ingle, K.K.; Gandhi, V.; Keefe, F.J.; Somers, T.J. Predictors of intervention session completion in a randomized clinical trial of a behavioral cancer pain intervention. *J Pain Symptom Manage* **2020**, *59*, 1268-1277, doi:http://dx.doi.org/10.1016/j.jpainsymman.2020.01.020.
20. Walle, T. NCT MOBILE - smartphone-based video consultations in medical oncology. http://www.drks.de/DRKS00015788 [cited 2020 Oct 29]. **2018**, doi:http://www.who.int/trialsearch/Trial2.aspx?TrialID=DRKS00015788.

21. Walle, T.; Erdal, E.; Mühlsteffen, L.; Singh, H.M.; Gnutzmann, E.; Grün, B.; Hofmann, H.; Ivanova, A.; Köhler, B.; Korell, F.; et al. Completion rate and impact on physician-patient relationship of video consultations in medical oncology: A randomized controlled open label trial. http://dx.doi.org/10.1136/esmoopen-2020-000912 *ESMO Open* **2020**, *5*, e000912.
22. Beaver, K.; Williamson, S.; Sutton, C.; Hollingworth, W.; Gardner, A.; Allton, B.; Abdel-Aty, M.; Blackwood, K.; Burns, S.; Curwen, D.; et al. Comparing hospital and telephone follow-up for patients treated for stage-I endometrial cancer (ENDCAT trial): A randomised, multicentre, non-inferiority trial. *BJOG* **2017**, *124*, 150-160, doi:10.1111/1471-0528.14000.
23. Dixon, P.; Beaver, K.; Williamson, S.; Sutton, C.; Martin-Hirsch, P.; Hollingworth, W. Cost-consequence analysis alongside a randomised controlled trial of hospital versus telephone follow-up after treatment for endometrial cancer. *Appl Health Econ Health Policy* **2018**, *16*, 415-427, doi:http://dx.doi.org/10.1007/s40258-018-0378-6.
24. Beaver, K.; Williamson, S.; Sutton, C.J.; Gardner, A.; Martin-Hirsch, P. Endometrial cancer patients' preferences for follow-up after treatment: A cross-sectional survey. *Eur J Oncol Nurs* **2020**, *45*, N.PAG-N.PAG, doi:http://dx.doi.org/10.1016/j.ejon.2020.101722.
25. Viers, B.R.; Lightner, D.J.; Rivera, M.E.; Tollefson, M.K.; Boorjian, S.A.; Karnes, R.J.; Thompson, R.H.; O'Neil, D.A.; Hamilton, R.L.; Gardner, M.R.; et al. Efficiency, satisfaction, and costs for remote video visits following radical prostatectomy: A randomized controlled trial. *Eur Urol* **2015**, *68*, 729-735, doi:http://dx.doi.org/10.1016/j.eururo.2015.04.002.
